# Dose-Response Relationship Between Exercise Duration and Enhanced Function and Cognition in Acutely Hospitalized Older Adults: A Secondary Analysis of a Randomized Clinical Trial

**DOI:** 10.1093/geroni/igae053

**Published:** 2024-06-01

**Authors:** Mikel L Sáez de Asteasu, Nicolás Martínez-Velilla, Fabricio Zambom-Ferraresi, Arkaitz Galbete, Robinson Ramírez-Vélez, Eduardo L Cadore, Pedro Abizanda, Javier Gómez-Pavón, Mikel Izquierdo

**Affiliations:** Navarrabiomed, Hospital Universitario de Navarra (HUN)-Universidad Pública de Navarra (UPNA), IdiSNA, Pamplona, Spain; CIBER of Frailty and Healthy Aging (CIBERFES), Instituto de Salud Carlos III, Madrid, Spain; Navarrabiomed, Hospital Universitario de Navarra (HUN)-Universidad Pública de Navarra (UPNA), IdiSNA, Pamplona, Spain; CIBER of Frailty and Healthy Aging (CIBERFES), Instituto de Salud Carlos III, Madrid, Spain; Navarrabiomed, Hospital Universitario de Navarra (HUN)-Universidad Pública de Navarra (UPNA), IdiSNA, Pamplona, Spain; CIBER of Frailty and Healthy Aging (CIBERFES), Instituto de Salud Carlos III, Madrid, Spain; Navarrabiomed, Hospital Universitario de Navarra (HUN)-Universidad Pública de Navarra (UPNA), IdiSNA, Pamplona, Spain; Navarrabiomed, Hospital Universitario de Navarra (HUN)-Universidad Pública de Navarra (UPNA), IdiSNA, Pamplona, Spain; CIBER of Frailty and Healthy Aging (CIBERFES), Instituto de Salud Carlos III, Madrid, Spain; Exercise Research Laboratory, School of Physical Education, Physiotherapy and Dance, Universidade Federal do Rio Grande do Sul, Porto Alegre, Brazil; CIBER of Frailty and Healthy Aging (CIBERFES), Instituto de Salud Carlos III, Madrid, Spain; Department of Geriatrics, Complejo Hospitalario Universitario de Albacete, Albacete, Spain; Department of Geriatrics, Hospital Central de la Cruz-Roja, San José y Santa Adela, Universidad Alfonso X el Sabio, Madrid, Spain; Navarrabiomed, Hospital Universitario de Navarra (HUN)-Universidad Pública de Navarra (UPNA), IdiSNA, Pamplona, Spain; CIBER of Frailty and Healthy Aging (CIBERFES), Instituto de Salud Carlos III, Madrid, Spain

**Keywords:** Hospitalization, Multicomponent training, VIVIFRAIL

## Abstract

**Background and Objectives:**

Exercise may reverse functional decline in hospitalized older adults, but the optimal duration is unclear. This study examined the potential relationship between in-hospital multicomponent exercise program duration and changes in physical function, cognition, and muscle function to maximize exercise-related health benefits in acutely hospitalized older patients.

**Research Design and Methods:**

This secondary analysis of a multicenter randomized controlled trial examined the relationship between the duration of an in-hospital multicomponent exercise program and changes in physical function, cognition, and muscle strength in 570 acutely hospitalized older adults. Participants completed 3, 4, or 5–7 consecutive days of exercise based on the progression of their acute medical illness. The acute clinical condition of the older patients was similar across the study groups (i.e., 3/4/5–7 days) at admission. Outcomes included the Short Physical Performance Battery (SPPB) for functional capacity, Gait Velocity Test for gait speed, handgrip for muscle strength, and cognitive tests.

**Results:**

Of the 570 patients included in the analysis, 298 were women (52.3%), and the mean (*SD*) age was 87.3 (4.8) years. Exercise groups increased SPPB scores compared with controls, with gains of 1.09 points after three days, 1.97 points after four days, and 2.02 points after 5–7 days (*p* < .001). The 4-day program showed the most significant benefit for functional capacity. Gait velocity increased by 0.11 m/s after 4 and 5–7 days (*p* = .032). Similar dose-response relationships were seen for handgrip strength and cognition, with 5–7 days showing more significant gains than three days (*p* < .05).

**Discussion and Implications:**

Multicomponent exercise programs enhance physical and cognitive function in hospitalized older adults, regardless of exercise dosage. A 4-day program significantly boosts functional capacity, although 5–7 days improves handgrip strength and cognition, highlighting the importance of exercise dosage in countering functional decline. Implementing evidence-based inpatient exercise prescriptions can help reverse muscle weakness and improve cognitive and physical function.

Clinical Trial Registration: NCT04600453

**Translational Significance:** Researchers identified a dose-response relationship between the duration of the exercise program and change in physical and cognitive functions, with four consecutive days of exercise being the optimal dosage for enhancing functional capacity. These findings emphasize the potential benefits of longer exercise durations on handgrip strength and cognitive function, underscoring the importance of tailored exercise interventions for older patients.

## Background and Objectives

Hazards of prolonged bed rest (BD) have been widely investigated in the literature (1). Acute medical illnesses and subsequent hospitalization are often sentinel events that lead to short and long-term disability, especially in older adults (2–5). Along with functional decline, acute care hospitalization also raises the risk of cognitive impairment and delirium episodes in older patients (6,7). Hospital-acquired disability (HAD) is independently linked to a range of adverse effects, such as more extended hospital stays, increased resource use, caregiver burden, institutionalization, and mortality (4).

Adverse health effects of hospitalization may be partly explained by the sudden reduction in physical activity, regardless of age (8). Low mobility and BD occur every day during hospitalization (9). They are significant contributors to in-hospital physical deterioration, causing a decline in daily living (ADL) activities and loss of muscle function. Previous research has demonstrated a rapid decrease of >10% in total lean leg mass and accelerated deterioration of maximal muscle strength and power after 6–10 days of in-hospital inactivity (10,11). These are robust predictors of physical functional performance in older adults (12). A recent meta-analysis has shown that BD periods in young and older adults cause relative loss of total body mass (3% after 14 days), muscle mass (5.5% after 14 days), and muscle function (i.e., muscle strength [14%–16% after five days] and power [9%–15% after 7–14 days]), indicating the detrimental effects of inactivity during hospitalization, especially in the oldest old (8).

In this regard, in-hospital physical exercise interventions and early rehabilitation programs have been proposed as the cornerstone of treatment to counteract the functional decline and cognitive impairment that often occur in older patients during acute hospitalization (13,14). Previous research has shown findings that multicomponent interventions, including gait retraining, balance, mobility, and resistance exercises, with a particular focus on muscle power training, result in changes in functional abilities in older patients admitted to the acute care for the elderly (ACE) unit (15–17). Despite numerous in-hospital exercise interventions being conducted in older adults, recently, few reports have evaluated different dose-response of supervised exercise training within a single large, well-controlled study on physical function in patients admitted to the ACE unit.

The present study aligns with the different therapeutic strategies developed to prevent the hazards of prolonged BD episodes during hospitalization in older adults. Consistent evidence supports the role of in-hospital physical exercise in older adults. Still, more research is needed to consider that exercise prescriptions should be personalized, like other medical treatments in this population (18). Although the dose-response of resistance training is well-established in healthy older adults (18), the lack of investigation on this issue during acute hospitalization precludes understanding what exercise dose is necessary and optimal to prevent HAD, especially in older adults.

Therefore, the main aim of this study was to examine the potential relationship between in-hospital multicomponent exercise dosage and changes in physical function, cognition, and muscle function to maximize exercise-related health benefits in acutely hospitalized older patients.

## Research Design and Methods

### Design

A complete description of the study design and methods has been published (16), and this study represents a secondary analysis of a multicenter randomized controlled trial (RCT). The data collection took place from February 1, 2015, to April 1, 2021, in ACE units at three tertiary hospitals in Spain (Hospital Universitario de Navarra of Pamplona, Hospital Central de la Cruz Roja of Madrid, and Complejo Hospitalario Universitario of Albacete) (15,16). This study adhered to the Consolidated Standards of Reporting Trials (CONSORT) guideline (**S1 CONSORT Checklist**). Admissions to the ACE units were primarily due to heart failure, with heart failure pulmonary and infectious diseases from the accident and emergency department.

The multicenter RCT adhered to the principles of the Declaration of Helsinki (19) and was approved by the local ethics committee (Pyto 2018/7). All patients or their legal representatives provided written informed consent.

### Participants

All patients admitted to the ACE units were evaluated by geriatricians to determine potential eligibility for the RCT. The inclusion criteria were as follows: age ≥75 years, Barthel Index score ≥60 points (measured two weeks before admission), ability to ambulate (with/without assistance), and ability to communicate and cooperate with the research team. Exclusion criteria included expected hospital stay of <6 days, very severe cognitive decline (i.e., Global Deterioration scale score = 7), terminal illness, uncontrolled arrhythmias, acute pulmonary embolism, recent myocardial infarction, recent major surgery, or extremity bone fracture in the past three months.

### Randomization and Blinding

After the baseline assessment, participants were randomly assigned to either the intervention or control group using an online system generated by a statistician not involved in the study (http://www.randomizer.org). The assessment staff was blinded to the study design and group allocation. Although it was impossible to blind the participants, they were explicitly informed and reminded not to discuss their randomization assignment with the assessment staff to prevent bias.

### Intervention

During hospitalization, acutely hospitalized older patients meeting the inclusion criteria were randomly assigned to either the intervention or control (usual care) group in the first 48 hours after hospital admission. Following the baseline assessment, participants completed three, four, or between 5 and 7 days of intervention. For this study, participants were stratified into dosing groups (3, 4, or 5–7 days). Patients were not randomized to the duration of exercise (3, 4, or 5–7 days). Instead, the duration was determined by the patient’s length of stay in the hospital, which was influenced by the course of their acute medical illness. This stratification was based on the acute clinical condition of the older patients, which was similar at admission across study groups. Intervention details are summarized in Table 1a. A detailed description has been previously published (16).

**Table 1.** Intervention and Control Group Protocols

Session	Exercise	Day 1	Day 2	Day 3	Day 4	Day 5*	Day 6*	Day 7^a^
*Intervention group*								
Morning	Rises from a chair	1 × 5	1 × 10	2 × 10	3 × 10	3 × 8	3 × 8	3 × 8
	Leg press	1RM	2 × 10 (30% 1RM)	3 × 10 (40% 1RM)	3 × 10 (50% 1RM)	3 × 8 (60% 1RM)	3 × 8 (60% 1RM)	3 × 8 (60% 1RM)
	Chet press	1RM	2 × 10 (30% 1RM)	3 × 10 (40% 1RM)	3 × 10 (50% 1RM)	3 × 8 (60% 1RM)	3 × 8 (60% 1RM)	3 × 8 (60% 1RM)
	Leg extension	1RM	2 × 10 (30% 1RM)	3 × 10 (40% 1RM)	3 × 10 (50% 1RM)	3 × 8 (60% 1RM)	3 × 8 (60% 1RM)	3 × 8 (60% 1RM)
Evening	Leg extension (0,5–1,0 kg)		2 × 10	2 × 10	2 × 10	2 × 10	2 × 10	2 × 10
	Leg flexion (0,5–1,0 kg)		2 × 10	2 × 10	2 × 10	2 × 10	2 × 10	2 × 10
	Hip abduction (0,5–1,0 kg)		2 × 10	2 × 10	2 × 10	2 × 10	2 × 10	2 × 10
	Hand grip ball		2 × 10	2 × 10	2 × 10	2 × 10	2 × 10	2 × 10
*Control group*								
Morning	Rises from a chair	1 × 5	HUC^b^	HUC	HUC	HUC	HUC	HUC
	Leg press	1RM	HUC	HUC	HUC	HUC	HUC	HUC
	Chet press	1RM	HUC	HUC	HUC	HUC	HUC	HUC
	Leg extension	1RM	HUC	HUC	HUC	HUC	HUC	HUC

*Notes*: HUC = hospital usual care; 1RM = 1 repetition maximum.

^a^In case the patient is still hospitalized.

^b^Hospital usual care included verbal encouragement by geriatricians to reduce low-mobility episodes and to promote early mobilization.

#### Control group.

Participants in the usual-care group received standard hospital care, which included physical rehabilitation when necessary.

#### Intervention group.

Participants participated in a 3- to 7-day exercise program consisting of 2 daily 20-minute sessions (morning and evening). Adherence to the program was documented in a daily register, with a session considered completed if 90% or more of the prescribed exercises were performed correctly. Patients and their families were familiarized with the protocol the day before the intervention began. The intervention was adapted from the Vivifrail multicomponent physical exercise program to prevent falls and weakness in older adults (20). The morning sessions involved supervised progressive resistance training, balance, and walking exercises tailored to the individual’s functional status using variable resistance training machines (Matrix; Johnson Health Tech and Exercycle S.L., BH group). Upper and lower body strengthening exercises were performed at a load equivalent to 30%–60% of the 1-repetition maximum (1RM), with 2–3 sets of 8–10 repetitions. For the 1RM test, each participant´s maximal load was determined in no more than five attempts, with a 3-minute recovery period between attempts. Participants were instructed to perform the concentric phase of the exercises at high speed for optimized muscle power output, with proper execution ensured. Balance and gait training exercises were prescribed, including semi-tandem foot standing, line walking, stepping practice, walking with small obstacles, proprioceptive exercises on unstable surfaces, alteration of the base of support, and weight transfer from one leg to another.

In the evening, functional unsupervised exercises using light loads (0.5–1 kg anklets and handgrip ball), such as knee extension/flexion, hip abduction, and daily walking in the corridor of the ACE unit, were performed. The duration of the exercises was based on the clinical physical exercise guide “Vivifrail” (20).

The intervention was started when the geriatrician deemed the patient’s hemodynamic situation acceptable and when the patient could cooperate. A personalized diary was used to record the number of exercise sessions completed during the afternoon session in the hospital. The endpoints were evaluated at the start of the intervention and again on the day of discharge.

In the researcher’s study, the safety and well-being of the researcher’s participants were of paramount importance. Each session was systematically monitored for signs of excessive strain, such as undue fatigue, discomfort, or pain. Qualified fitness professionals, specialized in geriatric exercise protocols, supervised all sessions, adjusting activities to accommodate individual participant needs. A strict participant-to-provider ratio was enforced to guarantee personalized supervision. Additionally, comprehensive safety measures included preliminary health screenings to rule out exercise contraindications, continuous vital sign monitoring, and immediate access to emergency medical equipment. Participants were also instructed to identify and promptly report any potential concerns during exercise sessions. Based on the multicomponent guide “Vivifrail” (20), the exercise program was carefully structured to escalate intensity in a controlled manner individually. This tailored, gradual approach ensured participants could adapt safely to the increasing exercise demands. The exercise selection and intensity levels were informed by established, evidence-based protocols tailored to the older adult demographic to maximize safety and effectiveness.

### Endpoints

The primary aim of the parent trial was to assess the impact of an in-hospital multicomponent exercise program on the physical function of acutely hospitalized older adults (16). For the secondary analysis presented in this manuscript, the primary endpoint was to examine the dose-response relationship between exercise duration and the change in functional capacity using the Short Physical Performance Battery (SPPB). The SPPB test comprises the usual walking speed over 4 m, a balance test, and the Five Times Sit to Stand Test. The sum of the scores obtained in these tests determines the final SPPB score, which ranges from 0 (worst) to 12 (best) (21). The meaningful change has been 1 point for the SPPB (22).

A 6-m Gait Velocity Test (GVT) was used as a secondary endpoint to measure functional changes. For the GVT, the patients were instructed to walk at their self-selected usual pace on a smooth, horizontal walkway, and a change of 0.1 m/s during hospitalization was considered a meaningful change (22). Isometric handgrip strength was also measured using a handheld dynamometer (T.K.K. 5401 Grip-D, Japan), and the highest value obtained from two valid trials was used for analysis. The clinically meaningful change for handgrip strength was considered 1 kg (23). Finally, changes in cognitive function were evaluated using the Mini-Mental State Examination (MMSE) (24), which is a 30-point questionnaire that ranges from 0 (worst) to 30 (best). The meaningful change for the MMSE was established at 3 points (25).

### Statistical Analysis

Statistical power considerations were reported previously (16). Researchers used the intention-to-treat approach. Baseline differences between the intervention and the control group were assessed using means and standard deviation for continuous variables and via frequencies and percentages for the categorical ones.

To evaluate the effect of the intervention on each group of study days (i.e., three days, four days, and five or more days), linear mixed models were fitted for each clinical outcome variable, stratified by study-day group. In each model, researchers included randomization group, time, and group-by-time interaction as fixed effects and participants as random effects. Time was treated as a categorical variable. To compare the intervention effects of the three study-day groups, a linear mixed model was fitted with the whole sample, including participants as a random effect and randomization group, time, study-day group, and their two and 3-way interactions as fixed effects, using the latter to evaluate the differences. Center was included as a randomized effect in the linear mixed model in a preliminary analysis, but researchers did find differences between centers. Thus, it was decided that the results of this study should not be adjusted by the center. Researchers calculated corresponding 95% confidence intervals (95% CI) and *p* values for three prespecified intergroup contrasts within each group over time, and researchers examined distributions of residuals. Forest plots of Cohen’s *d* effect sizes (ES) and corresponding 95% CI for the study-day group's physical function, muscle strength, and cognition endpoints were pooled through random-effects meta-analysis models, using the command “metan.” Heterogeneity was reported through the Higgins’ *I*^2^ statistic. To make inferences about the magnitude of the effect for groups and dose effects, effect sizes (ES) were calculated (26) and expressed as 90% confidence limits (27). The following formula was used to calculate the ES: [(postmean − premean)/SDpooled], where SDpooled = √ [(SDpre2 + SDpost2)/2] (26). An ES of <0.2 was considered trivial, 0.2–0.6 small, 0.6–1.2 moderate, 1.2–2.0 large, and 2.0–4.0 very large (28). Data were analyzed using SPSS-IBM (Software, v.21.0 SPSS Inc., Chicago, IL, USA) and R version 4.1.0. The significance level was set at α=0.05, and all tests performed were two-tailed.

## Results

**Figure 1.**
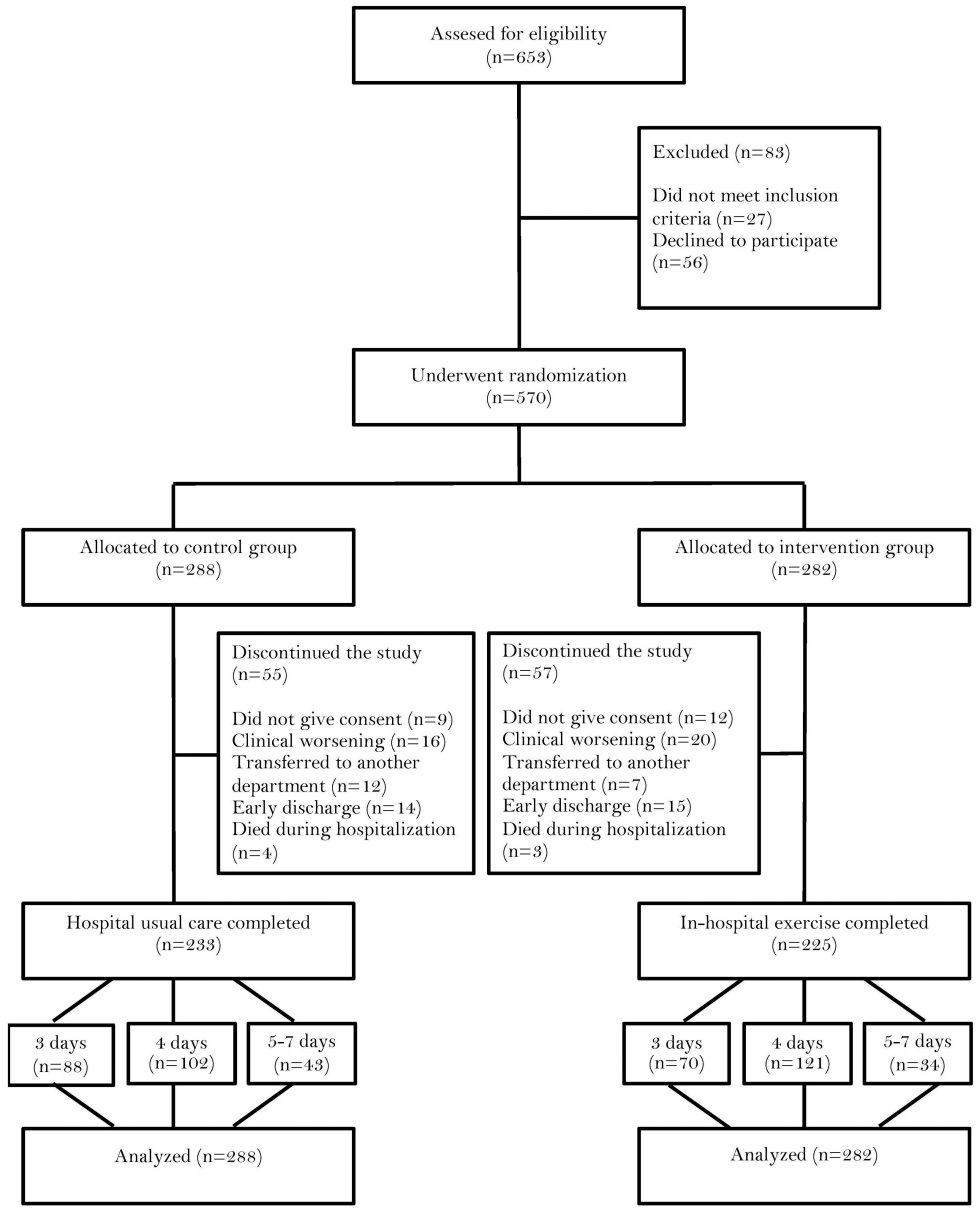
Study flow diagram.

The study flow diagram is shown in Figure 1. At baseline, there were no relevant differences between groups regarding demographic and clinical characteristics or study endpoints. The Researcher’s analysis included 570 patients, of whom 298 were women (52.3%). The mean age of the patients was 87.3 (4.8) years, with a range of 75–101 years. Among the patients, 193 patients (33.9%) were nonagenarians. The median length of hospital stay was eight days for both groups (interquartile range, three days for the control group, and four for the intervention group, respectively). These findings are summarized in Table 2.

**Table 2.** Clinical and Demographic Characteristics of the Participants

Variable	Control group (*n* = 288)	Intervention group (*n* = 282)
*Demographic data*		
Age, years, *M* (SD)	87.3 (5.1)	87.4 (4.6)
Women, *N* (%)	157 (54)	141 (50)
Body mass index, kg/m^2^, *M* (SD)	26.9 (5.0)	27.0 (4.8)
*Clinical data, M (SD)*		
Barthel Index, score	87.2 (12.0)	88.1 (11.8)
CIRS score, median (IQR)	13.0 (7)	12.0 (7)
MNA score, median (IQR)	18.0 (14)	18.0 (13)
1RM leg press, kg	60.4 (33.6)	56.0 (29.5)
1RM chest press, kg	25.2 (11.7)	24.5 (11.4)
1RM knee extension, kg	39.7 (17.8)	37.9 (14.5)
GDS, score	3.6 (2.9)	3.7 (2.6)
QoL (EQ-VAS), score	62.3 (22.9)	62.2 (23.8)
*Endpoint measures, M (SD)*		
SPPB, score	4.7 (2.8)	4.6 (2.7)
GVT, m/s	0.5 (0.2)	0.5 (0.2)
MMSE, score	23.0 (4.3)	22.3 (4.9)
Handgrip strength, kg	16.7 (7.1)	17.1 (6.6)
*Admission reason, N (%)*		
Cardiovascular	93 (32)	92 (33)
Infectious	74 (26)	70 (25)
Pulmonary	34 (12)	33 (12)
Gastrointestinal	26 (9)	25 (9)
Neurological	15 (5)	15 (5)
Other	46 (16)	47 (16)

Data are mean (*SD*) unless otherwise stated.

*Notes*: 1RM = 1-repetition maximum; CAM= Confusion Assessment Method; CIRS= Cumulative Illness Rating scale; GDS= Yesavage Geriatric Depression scale; GVT= Gait Velocity Test; IQR= interquartile range; MNA= Mini-nutritional Assessment; MMSE= Mini-Mental State Examination; QoL= quality of life; EQ-VAS = visual analog scale of the EuroQol questionnaire (EQ-5D); SPPB = Short Physical Performance Battery.

Out of the 570 participants, 158 older patients completed three days of intervention (88 in the control group and 70 in the intervention group), although 223 completed four days of intervention (102 in the control group and 121 in the intervention group). Additionally, 77 participants completed between 5 and 7 days of intervention, with 43 in the control group and 34 in the intervention group. In the control group, 20 participants received physical rehabilitation as part of the hospital's usual treatment. The drop-out rate was 19.65%. Researchers found no adverse effects associated with the prescribed exercises, and no patient had to interrupt the intervention or modify their hospital stay because of it.

**Figure 2.**
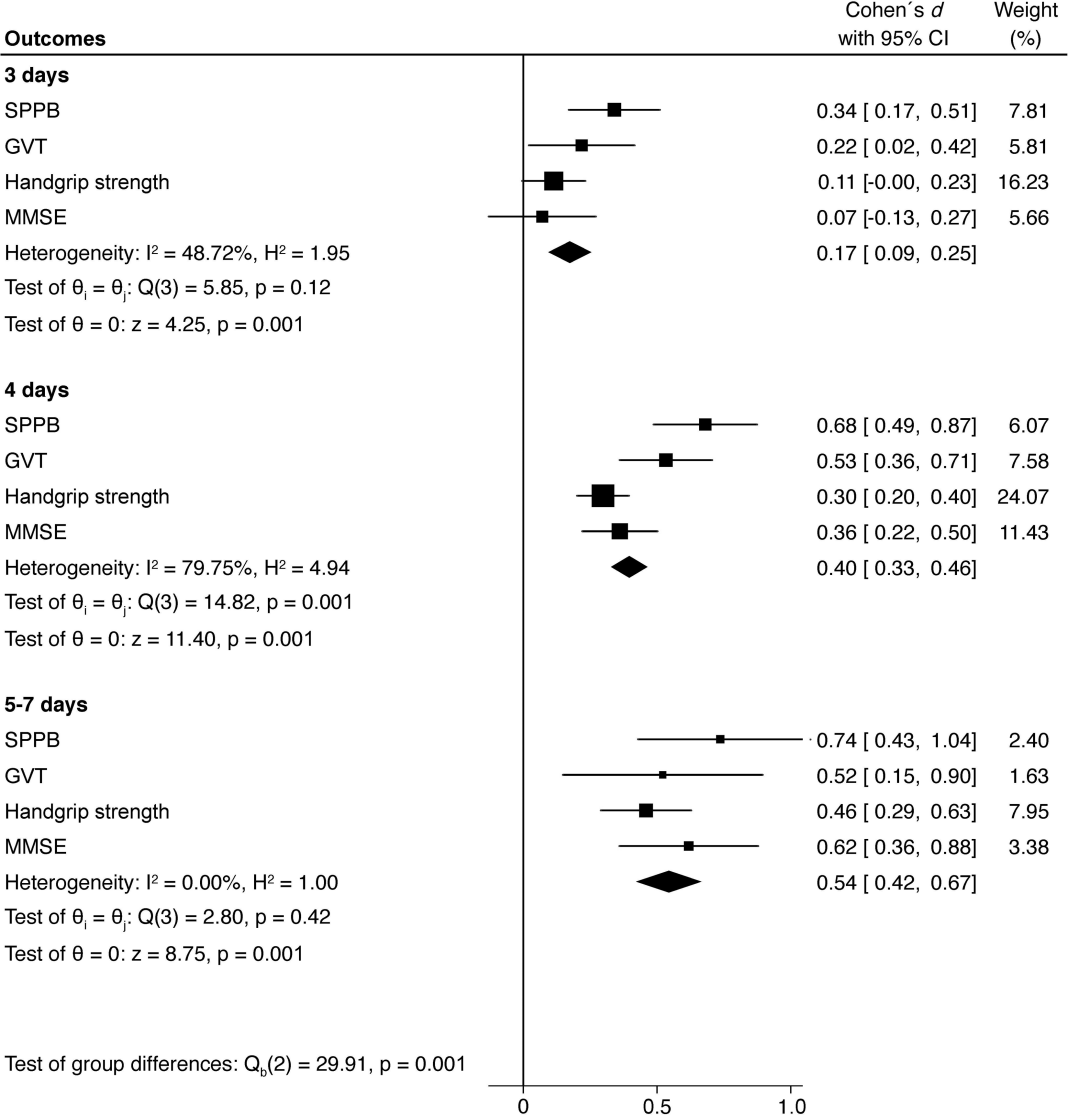
Forest plot of Cohen´s de-effect effect sizes and corresponding 95% confidence intervals for physical function, muscle strength, and cognition for the 3-day, 4-day, and 5–7-day intervention groups. Notes: CI = Confidence Interval; GVT = Gait Velocity Test; MMSE = Mini-Mental State Examination; SPPB = Short Physical Performance Battery.

The primary analysis showed that exercise intervention provided significant benefits over usual care, regardless of the number of days of training (i.e., 3, 4, or between 5 and 7 days) on the SPPB scale (all *p* < .001). At discharge, the exercise group that received three days of training showed a mean increase of 1.09 points (95% CI: 0, 56 to 1.62 points with ES small = 0.34), the group that received four days of training 1.97 points (95% CI: 1.43–2.50 points with ES moderate = 0.68), and the group that received between 5 and 7 days of training showed a mean increase of 2.02 points (95% CI: 1.25–2.78 points with ES moderate = 0.74) over the control group. Notably, there was almost 1-point of functional improvement, which is considered a meaningful clinical change for the SPPB, in the 4-day and the 5–7-day exercise groups compared with the 3-day training group, with significant differences observed in the 4-day exercise group (Table 3 and Figure 2). Researchers found substantial changes in GVT of 0.11 m/s (95% CI: 0.08, 0.14 with ES small = 0.53) in the 4-day and 0.11 m/s (95% CI: 0.05, 0.18 with ES small = 0.52) in the 5–7-day group at discharge compared with the usual care group. However, researchers did not find significant between-group differences after three days of intervention, with a mean change of 0.04 m/s (95% CI: −0.02 to 0.10 with ES small = 0.22). Additionally, researchers observed GVT gains in the 4-day and the 5–7-day exercise groups compared with the 3-day training group, with significant differences observed in the 4-day exercise group (0.07 m/s; 95% CI: 0.01–0.13; Table 3 and Figure 2).

**Table 3.** Results of Endpoints by Group and Days of Study

Endpoint	Control group		Intervention group		Between-group differences		Between study day group differences	
	Mean change (95% CI)	*p*-Value	Mean change (95% CI)	*p*-Value	Mean change (95% CI)	*p*-Value	Mean change (95% CI)	*p*-Value
*SPPB*								
Three days	0.23 (−0.12, 0.58)	.193	1.32 (0.93, 1.73)	<.001	1.09 (0.56, 1.62)	<.001	ref	
Four days	0.50 (0.10, 0.90)	.014	2.47 (2.10, 2.83)	<.001	1.97 (1.43, 2.50)	<.001	0.87 (0.10, 1.63)	.028
5-7 days	−0.13 (−0.65, 0.38)	.616	1.88 (1.32, 2.44)	<.001	2.02 (1.25, 2.78)	<.001	0.91 (−0.11, 1.94)	.083
*GVT*								
Three days	0.03 (−0.01, 0.07)	.124	0.07 (0.03, 0.12)	.002	0.04 (−0.02, 0.10)	.178	ref	
Four days	0.01 (−0.01, 0.04)	.253	0.12 (0.10. 0.15)	<.001	0.11 (0.08, 0.14)	<.001	0.07 (0.01, 0.13)	.032
5–7 days	−0.01 (−0.05, 0.03)	.679	0.10 (0.05, 0.15)	<.001	0.11 (0.05, 0.18)	.001	0.07 (−0.01, 0.16)	.107
*Handgrip*								
3 days	−0.15 (−0.74, 0.43)	.608	0.94 (0.29, 1.60)	.005	1.10 (0.22, 1.97)	.015	ref	
Four days	−0.45 (−0.89, −0.02)	.043	1.40 (1.01, 1.79)	<.001	1.85 (1.26, 2.44)	<.001	0.75 (−0.23, 1.74)	.137
5–7 days	−1.00 (−1.65, −0.36)	.003	1.79 (1.09, 2.49)	<.001	2.79 (1.84, 3.74)	<.001	1.69 (0.38, 3.01)	.012
*MMSE*								
Three days	0.65 (0.01, 1.30)	.048	1.04 (0.30, 1.77)	.006	0.38 (−0.59, 1.36)	.444	ref	
Four days	0.50 (0.07, 0.92)	.024	2.07 (1.69, 2.44)	<.001	1.57 (1.00, 2.14)	<.001	1.20 (0.15, 2.24)	.026
5–7 days	−0.58 (−1.28, 0.12)	.111	2.19 (1.38, 3.01)	<.001	2.77 (1.70, 3.85)	<.001	2.39 (1.00, 3.79)	<.001

*Notes*: CI = confidence interval; GVT = Gait Velocity Test; MMSE = Mini-Mental State Examination; SPPB = Short Physical Performance Battery.

Data are expressed as changes from baseline (admission) to discharge. For differences between study-day groups, the 3-day group was used as a reference for the *p*-value calculation.

Researchers observed significant differences between groups in muscle function as assessed by handgrip strength, regardless of days of training (*p* < .01). At discharge, the 3-day exercise group showed a significantly more significant change of 1.10 kg (95% CI: 0.22–1.97 kg with ES trivial = 0.11), the 4-day exercise group showed a significant more remarkable change of 1.85 kg (95% CI: 1.26–2.44 kilograms with ES small = 0.30) and the 5–7 day exercise group showed a significant more remarkable change of 2.79 kg (95% CI: 1.84–3.74 kilograms with ES small = 0.46) compared with the usual care group. Moreover, significant differences were obtained between the 5–7-day exercise and 3-day training groups, with a clinically meaningful improvement of 1.69 kg (95% CI: 0.38–3.01; Table 3 and Figure 2).

In terms of cognitive function, the difference in change from baseline to discharge of the MMSE was significantly higher in the intervention group compared with the control group (1.57 points; 95% CI: 1.00–2.14 points with ES small = 0.36 for the 4-day group, and 2.77 points; 95% CI: 1.70–3.85 points with ES moderate = 0.62 for the 5–7-day group, respectively). Nevertheless, researchers did not find significant between-group differences after three days of intervention, with a mean change of 0.38 points (95% CI: −0.59 to 1.36 with ES trivial = 0.08). Notably, significant differences were observed between the 4-day (1.20 points; 95% CI: 0.15–2.24) and 5–7-day exercise groups (2.39 points; 95% CI: 1.00–3.79) compared with the 3-day intervention group, indicating cognitive gains after more days of exercise training (Table 3 and Figure 2). As shown in Figure 2, the overall ES was higher in the 5–7-day exercise group, and significant differences were obtained in this group compared with the 3-day (*p* < .001) and 4-day intervention groups (*p* < .038). Significant differences were also observed between the 3-day and 4-day exercise groups (*p* < .001).

**Figure 3.**
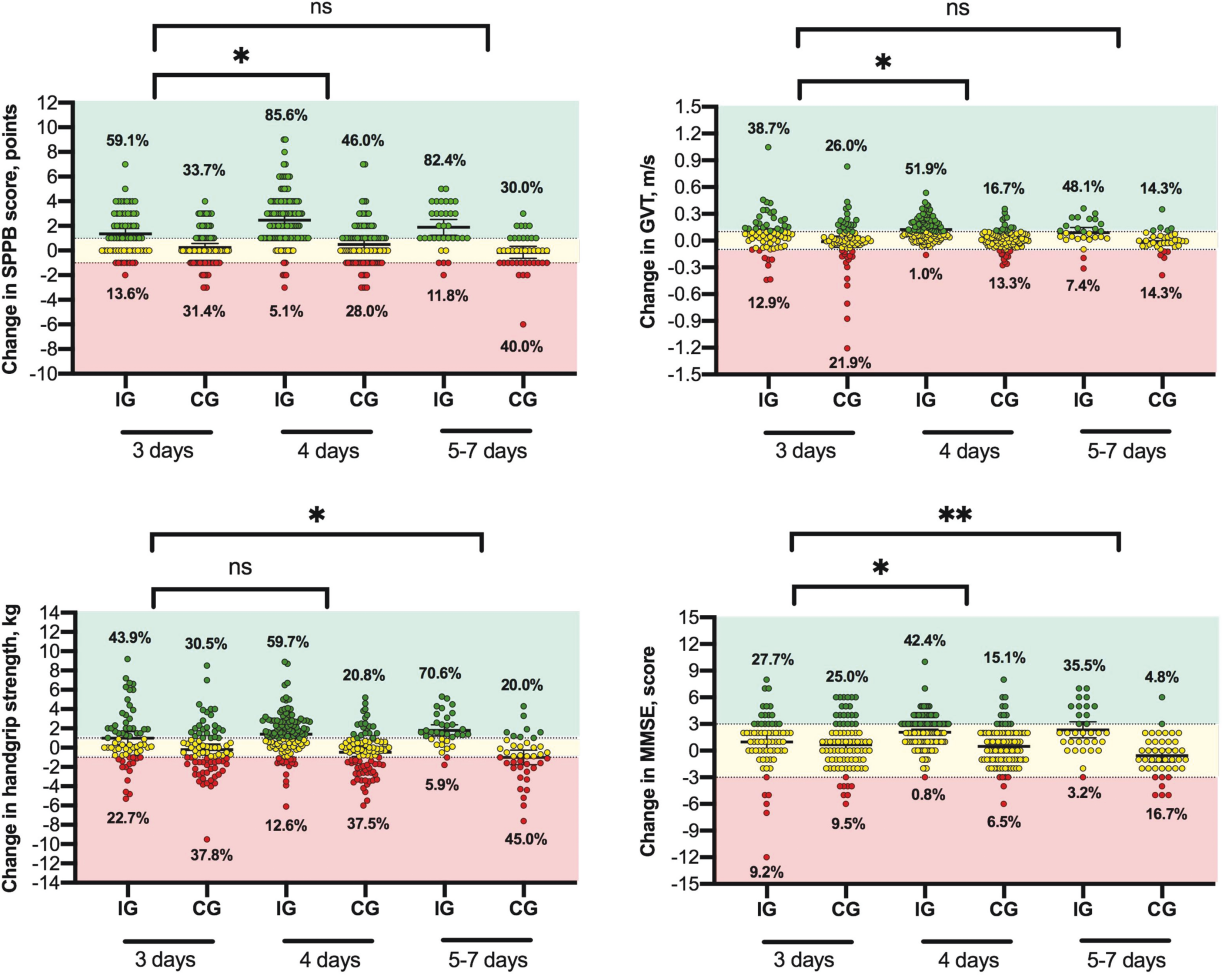
Changes in within-group punctuation in the Short Physical Performance Battery (SPPB), Gait Velocity Test (GVT), handgrip strength, and Mini-Mental State Examination (MMSE). The horizontal dashed lines indicate the median and Q1 and Q3. *p < .05; **p < .001 in linear mixed model analysis.

Finally, the percentage distribution of patients with improvements (i.e., considering the meaningful clinical change), no changes or worsening from admission to discharge notably differed between the intervention and the control groups, indicating a beneficial effect of physical exercise for all endpoints (Figure 3).

## Discussion and Implications

The primary finding of this prospective, randomized, controlled exercise trial is a strong relationship between exercise dose (i.e., three days, four days, and between 5 and 7 days) and improvements in physical function, muscle strength, and cognition in acutely hospitalized older adults. Although logical, this finding has not been previously reported in adequately powered studies with a tightly controlled exercise dose that evaluated the relationship between exercise dose and health-related benefits when treating older patients admitted to ACE units.

Acute hospitalization often leads to a sudden reduction in physical activity levels, resulting in muscle function loss not only due to unloading but also by triggering muscle denervation (28) and neuromuscular alterations (8,29). Previous studies have identified physical inactivity (or bed rest) as a significant contributing factor to HAD in older patients (9). The findings suggest that short hospital stays may prevent functional decline, but longer stays may lead to developing HAD in control patients. In-hospital mobility programs that only involve ambulation and behavioral strategies to enhance activities of daily living do not seem to have functional benefits for acutely hospitalized older adults (30). Therefore, consistent with previous research, the study suggests that interventions beyond walking stimulation are necessary to improve physical function in acutely hospitalized older adults.

In-hospital exercise intervention has been proposed as a cornerstone treatment to address the functional and cognitive impairment that often occurs during hospitalization (13,14). The study shows that multicomponent exercise interventions, focusing on progressive strengthening training, offer significant improvements in functional, cognition, and muscle function benefits compared to standard hospital care. The anti-inflammatory role of in-hospital physical exercise may be one of the underlying mechanisms for these positive changes in functional, cognitive, and muscle strength outcomes in this population (31,32).

The study also investigated the relationship between exercise dosage (i.e., 3, 4, or 5–7 days) and changes in functional, cognitive, and muscle strength from admission to discharge. The acute clinical condition of the older patients was similar between study groups (i.e., 3, 4, or 5–7 days) at admission. However, hospitalized older adults completed 3, 4, or 5–7 days based on the course of their acute medical illness rather than randomization. Other clinical characteristics, such as functional status at admission, likely play a vital role in the trajectory of patients during hospital stays and may explain the exercise-induced benefits differences between study groups (33). Researchers found that older adults who completed a 4-day exercise program showed significantly higher functional gains than those who trained for three consecutive days. However, no significant differences in functional benefits were observed after more than four training days. Although three days of physical exercise improved physical function in most acutely hospitalized older adults, the findings suggest that a 4-day exercise program may be optimal for enhancing functional capacity. This information and other emerging evidence on exercise dosage should be considered when developing clinical recommendations for acute hospitalization periods.

In addition, the study showed that multicomponent exercise significantly benefits muscle function over habitual hospital care, regardless of the exercise dosage. Previous evidence has also demonstrated the effectiveness of exercise in reversing muscle function and improving physical performance deficits in acutely hospitalized older adults (11). The study also observed a relationship between exercise dose and muscle strength, with higher muscle strength improvements after 5–7 consecutive days of exercise training. Therefore, longer active hospital stays with individualized tailored multicomponent training that includes progressive resistance training may offer an opportunity for more significant muscle strength gains at discharge compared with shorter hospitalization periods. However, it is essential to consider the data from the usual-care group, as increasing days of hospitalization may also lead to muscle weakness, which is associated with a lower likelihood of survival after hospitalization in older patients (34).

The study demonstrated significant cognitive benefits associated with in-hospital exercise intervention. Previous research has also highlighted the effectiveness of physical exercise in improving cognitive function in very old patients during acute hospitalization, with multicomponent exercise training showing the most promising results (14,35). Furthermore, multicomponent exercise training, such as the one applied in this multicenter RCT, may have the most beneficial results (36). This study further supports these findings by showing a relationship between exercise dose and cognitive changes during hospitalization, providing consistent evidence of exercise’s positive effects on cognitive function. An earlier meta-analysis also suggested that exercise programs characterized by short session duration and high training frequency are most effective in improving cognitive function in older adults with cognitive impairment (37).

## Limitations

This study has limitations that should be acknowledged. It is a secondary analysis, and the primary analysis results were previously published (15,16). Although the parent trial was adequately powered for its primary endpoints, researchers must recognize that this secondary analysis was not independently powered but instead relied on the comprehensive data collected in the parent trial. Moreover, researchers have enriched the researcher’s manuscript with additional details on the medical diagnoses and complexities of the recruited cohorts to facilitate better judgment on the generalizability of the findings. Diagnoses were matched across control and intervention groups, ensuring that any observed effects could be attributed to the intervention rather than underlying medical variability. The generalizability of the results is limited as researchers only included a selected population with relatively good functional capacity before hospital admission (i.e., Barthel Index score ≥60 points), excluding older adults with unstable hemodynamic conditions or those who could not walk at admission. Future studies should investigate the optimal exercise protocol for improving physical function in older adults with a lower functional reserve and use more feasible materials for prescribing tailored resistance exercises, such as elastic bands. Furthermore, the 5–7-day group sample was smaller than the other groups, and no statistical adjustment was performed for the different centers involved in the study. The length of the hospital stay also limits the extrapolation of the dose-response relationship, as it reflects only a minimum exercise dose without determining if a plateau is reached beyond the length of the treatment period. Although an exclusion criterion for this study was an expected hospital stay of <6 days, researchers observed that many participants in the usual care and intervention groups were discharged before reaching six study days. This discrepancy highlights the inherent variability in the clinical course of acute medical conditions, which can lead to earlier discharges than initially anticipated. Such variability may have impacted the participants’ ability to fully engage in the exercise intervention, potentially influencing the study’s findings. Future research should consider more flexible inclusion criteria regarding hospital stay durations to accommodate such variability better and ensure a comprehensive assessment of the intervention’s effects. Finally, due to the nature of the intervention, it was impossible to blind physical therapists responsible for prescribing and supervising the intervention, patients, or families to the group assignment of a patient.

This study, however, has several strengths. It was a multicenter randomized controlled trial that aimed to examine the effectiveness of an in-hospital multicomponent exercise intervention on functional capacity and other health-related endpoints, such as cognition and muscle strength, in older adults admitted to ACE units. To the researcher’s knowledge, this is the first study to examine the effects of exercise dose-response on physical function in hospitalized patients of advanced age. Notably, the mean age of the participants was 87.3 (4.8) years (range 75–101 years, with 193 patients [33.9%] being nonagenarians). Considering the findings obtained in this study, it will be feasible to conduct adequate studies to evaluate how the findings can be generalized in this population and other hospitalized patients.

Moreover, the RCT included older adults with multiple comorbidities and mild or moderate dementia, who are frequently excluded from exercise studies. Although the immediate benefits of exercise interventions are evident, the persistence of these effects without continued activity remains uncertain, underscoring the need for future research to focus on designing and implementing effective post-discharge exercise continuation strategies to ensure the durability of health gains. Finally, the findings highlight the relevance of consideration of individual variability in response to exercise interventions among hospitalized older adults. Future studies should continue exploring personalized medicine approaches that tailor exercise regimens to individual patient profiles to achieve optimal outcomes (37).

## Conclusion

A tailored multicomponent exercise program improves functional capacity, cognition, and muscle function in acutely hospitalized older patients. Multicomponent in-hospital exercise exhibits beneficial effects regardless of exercise dosage and should be a standard part of acute care for older patients. The significance of the findings has important implications for the health care of hospitalized older patients, highlighting the potential benefits of exercise interventions for this population.

Furthermore, integrating patient education and self-management strategies into these exercise programs may provide a sustainable model for preserving the physical and cognitive benefits post-discharge. This approach could bridge the gap from hospital-based rehabilitation to long-term community-based support, encouraging ongoing engagement in health-promoting behaviors. In addition, establishing habitual physical activity and integrating lifestyle interventions are essential for maintaining health benefits post-discharge, necessitating robust follow-up programs and access to supportive community resources to ensure long-term success. This continuity is crucial for extending the life span and quality of health enhancements initiated during the hospital stay. Further research is needed to ascertain the transferability of the researcher’s results and the efficacy of innovative exercise protocols using fewer resources in ACE settings in other countries.

## Data Availability

Data, analytic methods, or materials are available on request from the Public University of Navarre by contacting Mikel Izquierdo (mikel.izquierdo@unavarra.es). The study reported in the manuscript was not preregistered.
